# Impact of type 2 diabetes mellitus on the prevalence of apical periodontitis in endodontically treated and untreated teeth

**DOI:** 10.25122/jml-2024-0330

**Published:** 2024-10

**Authors:** Andreea Marica, Razvan Chirla, Mihai Porumb, Lucian Roman Sipos, Raluca Ortensia Cristina Iurcov, Simona Cavalu

**Affiliations:** 1 Department of Doctoral Sciences, Faculty of Medicine and Pharmacy, University of Oradea, Oradea, Romania; 2 Department of Preclinical Sciences, Faculty of Medicine and Pharmacy, University of Oradea, Oradea, Romania; 3 Department of Dentistry, Faculty of Medicine and Pharmacy, University of Oradea, Oradea, Romania

**Keywords:** endodontics, root canal therapy, diabetes mellitus, apical periodontitis, glycemic control

## Abstract

Apical periodontitis (AP) is a common dental condition that can be influenced by diabetes mellitus. This study aimed to investigate the impact of type 2 diabetes mellitus (T2DM) on the prevalence and severity of AP, considering the adequacy of endodontic treatments. A total of 180 patients selected based on specific dental criteria from a private clinic in Oradea, Romania, were included in this study. Clinical data were collected through medical records and panoramic radiographs. Statistical analyses were conducted using SPSS software, employing the Fisher test, Mann-Whitney test, and binary logistic regression to determine correlations between T2DM and AP in both endodontically treated and non-treated teeth. Additionally, we examined the relationship between diabetes and AP in teeth that received adequate root canal treatment (RCT), as well as the correlation of AP with the adequacy of endodontic treatment. Non-treated teeth were significantly more likely to have AP in patients with T2DM than in non-diabetic patients (OR = 5.3, *P* < 0.001). No significant difference in AP prevalence was observed between treated teeth in diabetic and non-diabetic patients. Inadequate RCT was associated with a higher incidence of AP, regardless of diabetes status (OR = 26.9, *P* < 0.001). The study concludes that DM significantly increases the risk of AP in untreated teeth, with diabetic patients showing a higher prevalence of AP than non-diabetic patients. However, this increased risk is not observed in adequately treated teeth. The quality of RCT is critical, as inadequate RCT is linked to a higher incidence of AP, regardless of diabetes status.

## INTRODUCTION

Diabetes mellitus is a chronic, complex, and progressive systemic disease characterized by metabolic disorders affecting carbohydrate, lipid, and protein metabolism. It is primarily classified into two main types [1-6]. These disorders result from deficiencies in insulin secretion due to dysfunction of beta cells in the pancreas and/or resistance of the liver and muscles to insulin [7,8]. Diabetes mellitus (DM) is one of the most common metabolic conditions globally, significantly impacting overall health with no current cure, leading to increased morbidity and mortality rates [2,3].

The onset of diabetes is influenced by various factors, including genetic predisposition, environmental causes, obesity, and a lack of physical activity [8]. Type 1 diabetes mellitus (T1DM), also known as insulin-dependent diabetes, is characterized by an autoimmune reaction leading to the destruction of pancreatic beta cells, usually resulting in total insulin secretion loss [3,6]. Type 2 diabetes (T2DM) is more prevalent, accounting for approximately 90-95% of cases, and affects over 6% of the global population, typically presenting in adults aged 20 to 79 years [1,8].

In contrast to type 1 diabetes, T2DM is caused by insulin resistance along with insufficient insulin production to compensate, often linked to obesity, which contributes to insulin resistance by increasing circulating levels of free fatty acids derived from adipocytes, inhibiting glucose absorption, glycogen synthesis, and glycolysis [6]. Additionally, cells are unable to absorb and metabolize glucose due to the body’s inability to produce insulin or tissues' inadequate response to insulin, leading to increased blood glucose levels [8].

Diabetes mellitus affects the body's immune-inflammatory response, increasing patients' susceptibility to various oral conditions [5]. Common oral manifestations include periodontitis, oral candidiasis, oral cancer, oral potentially malignant disorders (OPMD), dental caries, burning mouth syndrome (BMS), salivary secretion alterations, taste perception alterations, halitosis, delayed wound healing [9]. This disease is associated with decreased quality of life, increased infection rates, and impaired wound healing, significantly impacting endodontic treatment [10].

Diabetes-induced hyperglycemia leads to difficulties in wound healing and systemic and oral manifestations. Studies indicated an association between uncontrolled diabetes mellitus and increased prevalence of periapical lesions, highlighting the negative effects of this condition on wound healing and immune function. Factors such as chronic inflammation, impaired leukocyte function, and changes in cytokine production contribute to the complications observed in patients with diabetes [4,5,10].

The objective of optimal glycemic control is essential to minimize the risks associated with diabetes mellitus and to improve the prognosis of endodontic treatment in these patients. Apical periodontitis is an inflammatory condition of the tissues surrounding the root apex of a tooth caused by bacterial infection of the endodontic system [5]. Periradicular lesions result from the irritation of periradicular tissues by polymicrobial irritants from the root canals of teeth with necrotic pulp [11].

The diagnosis of AP is established based on clinical and radiographic findings. Clinically, examinations may include sensitivity testing to cold, palpation, percussion, and identifying pain, swelling, or possible fistulas. Since chronic AP often lacks subjective symptoms such as pain, diagnosis based on radiographs becomes crucial.

Typically, in radiographs, AP is evident as a radiolucent area around the apex of the affected tooth. However, in the early stages of the disease, periapical tissue involvement may not always be detectable on radiographs [5,10,11]. Endodontic-periodontal lesions can be classified into the following categories: primary endodontic lesions, primary endodontic lesions with secondary periodontal complications, primary periodontal lesions, primary periodontal lesions with secondary endodontic complications, and true combined lesions [12]. Primary endodontic lesions should be treated with endodontic therapy first, as aggressive removal of periodontal ligament and cementum can negatively impact healing [13].

The effects of hyperglycemia on periapical status have been the subject of extensive research [1,14,15]. Increased blood glucose levels can trigger a series of abnormal molecular events leading to a disturbed inflammatory response, thus contributing to the onset of oral disorders associated with diabetes [1].

Several studies have reported an association between the prevalence of AP and DM. Patients with diabetes are more susceptible to developing oral diseases, including AP. Moreover, poorly controlled diabetes has been found to have a higher prevalence of periapical lesions during endodontic treatment [5]. Elevated blood glucose levels can promote abnormal molecular events leading to altered inflammatory responses, exacerbating AP and affecting healing [10].

Changes induced by diabetes mellitus in immune cell function lead to an inflammatory phenotype, manifested by increased production of proinflammatory cytokines in monocytes and polymorphonuclear leukocytes and decreased expression of growth factors in macrophages. These changes predispose to chronic inflammation, progressive tissue damage, and decreased tissue regeneration capacity [6].

The dental pulps of patients with diabetes often exhibit limited collateral circulation, impaired immunity, and an increased risk of pulp infections or necrosis. These aspects, along with dental pain and sporadic tendency towards ischemia-induced pulp necrosis, are important factors to consider in the dental management of patients with diabetes [10]. Additionally, chronic apical periodontitis maintains inflammation, potentially exacerbating diabetes control by increasing blood glucose levels and perpetuating an uncontrolled diabetic state [11].

Previous studies have shown that periodontal disease is associated with poorer glycemic control in patients with diabetes and an increased risk of diabetic complications. Diabetes treatment aims to maintain blood glucose within recommended limits, but the presence of periodontitis may influence this control. Additionally, diabetes does not cause root canal treatment failure but rather delays the healing process. Root canal therapy in patients with diabetes must be approached with caution, using controlled strategies to prevent microbial dissemination and promote proper healing. Patients with diabetes may have an increased rate of endodontic treatment failure and may require antibiotic therapy in certain situations [11]. These patients must benefit from sustained endodontic treatment supported by rigorous evaluations and effective antimicrobial regimens of the root canal [10].

Clinicians and patients need to be aware of the potential link between diabetes and the success of endodontic treatment and its impact on the prognosis of treated teeth. According to studies, diabetes predisposes to chronic inflammation, reduces tissue regeneration capacity, affects immune response by increasing susceptibility to infections, slows down wound healing, and is strongly associated with periodontal disease, which in turn is significantly linked to apical periodontitis and tooth loss, increasing the rate of endodontic retreatments [4,8,16]. Antibiotic therapy is justified in cases of urgency or situations of increased risk, such as acute oral infections or dentoalveolar surgical interventions [10]. Persistent microbial infection in the endodontic system causes post-treatment apical periodontitis. Other factors include extraradicular infection, extruded materials, and true cysts. The most common cause is residual microbes in the apical area [17]. In this context, the aim of our study was to investigate the impact of T2DM on the prevalence and severity of AP in both endodontically treated and untreated teeth. Specifically, we aimed to determine if diabetes influences the occurrence and healing of AP and how the adequacy of endodontic treatments affects these outcomes in patients with diabetes.

## MATERIAL AND METHODS

### Study design, inclusion, and exclusion criteria

**Figure 1:**
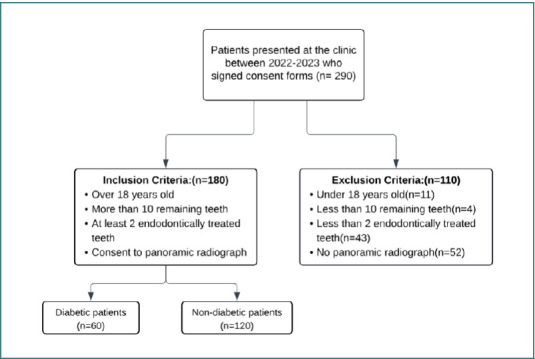
Flowchart of selection criteria

The participants for this study were selected from individuals who visited a private clinic in Oradea, Bihor County, Romania, for endodontic treatment between January 1st, 2022, and December 31st, 2023. The inclusion criteria required participants to be over 18 years old, have more than 10 remaining teeth, have at least 2 teeth that had previously undergone endodontic treatment, and either present with or consent to undergo a panoramic radiograph. The exclusion criteria were as follows: patients under 18 years old, patients with less than 10 remaining teeth, patients with fewer than 2 endodontically treated teeth, and patients who refused or did not undergo a panoramic radiograph. Initially, 290 patients were selected, but only 180 met the inclusion criteria (Figure 1).

Data were collected from the patient's consent forms, which included questions about their medical and dental history, lifestyle, and use of tobacco, alcohol, or drugs. Information about glycated hemoglobin (HbA1c) levels was obtained from the patient consent form, completed at the time of their presentation at the clinic. Patients with diabetes were asked to provide their most recent glycated hemoglobin measurement, taken within the past seven days, roughly coinciding with the date of the panoramic radiograph. After applying inclusion and exclusion criteria, patients were divided into two groups: the diabetic group, consisting of patients with T2DM with HbA1c levels ≥ 6.5%, and the control group, consisting of systemically healthy patients.

The patients' panoramic radiographs were digitized and analyzed individually by an endodontic specialist. Regarding the total number of teeth, irrecoverable root remnants were excluded from the total tooth count.

The periapical status was deemed normal and healthy in the presence of normal periapical structures and minor changes in bone structure. Thus, teeth were classified as healthy if the contour and width of the periodontal ligament space were normal or slightly widened around some overfilled root fillings, and the appearance of the surrounding bone was normal. The teeth were classified as pathological when changes in bone structure with mineral loss and periodontitis were observed, characterized by a well-defined radiolucent area. For multi-rooted teeth, the root exhibiting the most pronounced radiological pathology was considered for evaluation.

Our analysis covered endodontic treatments, considering only pulpectomies while excluding teeth with previously initiated therapy. According to the American Association of Endodontists (AAE), previously initiated therapy refers to partial endodontic treatment, such as pulpotomy. The next step was to analyze the presence or absence of AP according to the aforementioned criteria.

In evaluating the quality of endodontic treatments, treatments were deemed adequate when the root canals were obturated without radiologically visible voids in the gutta-percha mass, and the apical limit of the filling was located 0 to 2 mm from the radiographic apex. The root with the poorest treatment quality was used as the standard for multi-rooted teeth. Additionally, the number of these teeth presenting AP was noted. A separate analysis was performed to assess teeth affected by apical periodontitis that had not undergone prior endodontic treatment. These analyses aimed to observe how diabetes mellitus influences the occurrence of apical periodontitis and whether it affects healing in patients with radiologically adequate endodontic treatments.

### Statistical analysis

The statistical analysis was performed using the IBM SPSS Statistic Processor for Windows, version 22.0.0.0 (Armonk, NY: IBM). Correlations between categorical variables were assessed using the Fisher exact test, and odds ratios were calculated from binary logistic regression analyses. The Mann–Whitney test for independent samples was employed to compare the distributions of teeth displaying apical periodontitis.

## RESULTS

The demographic characteristics of the participants are presented in Table 1. Among the 180 patients (96 women and 84 men), 60 were diagnosed with type 2 diabetes mellitus (35 women and 25 men). The mean age of women was 48.7 ± 13.6 years and 45.8 ± 10.9 years for men, resulting in an overall mean age of 47.4 ± 12.5 years. The median age was 46.5 years for women and 44 years for men, resulting in an overall median age of 46 years.

**Table 1:** Demographic characteristics

Parameter	Total	Subcategories
Number of participants	180	Female (F) = 96 (53.3%) Male (M) = 84 (46.6%)
Diabetes group	60 (33.3%)	F = 35 (58.3%) M = 25 (41.6%)
Non-diabetes group	120 (66.6%)	F = 61 (50.8%) M = 59 (49.1%)
Mean age (years)	47.4 ± 12.5	F = 48.7±13.6 M = 45.8±10.9
Median age (years)	46.0	F = 46.5 M = 44.0

The correlation between AP, diabetes, and treatment adequacy was analyzed at the individual tooth and patient levels, with the results summarized in Table 2. A total of 4,439 teeth were studied, out of which 592 were with AP and 3,847 without AP. In addition, we also compared the statistical distributions of AP at the patient level in various histograms. Two patients without diabetes and one patient with diabetes did not have any non-treated teeth, and thus, they did not contribute to the statistical results presented in the corresponding histogram.

**Table 2:** Classification of teeth and patients by previous treatment, diabetes, and AP status

%	Treatment	Diabetes	With AP	Without AP	*P* value
Teeth (% of treated or untreated)	Non-treated	Yes	124 (3.6%)	865 (25%)	*P* < 0.001 (AP vs. diabetes)
		No	65 (1.9%)	2405 (69.5%)	
	Adequate RCT	Yes	22 (2.2%)	176 (18%)	*P* > 0.05 (AP vs. diabetes) *P* < 0.001 (AP vs. adequacy)
		No	67 (6.9%)	334 (34%)	
	Inadequate RCT	Yes	102 (10.4%)	34 (3.5%)	
		No	212 (21.6%)	33 (3.4%)	
Patients (% of treated or untreated)	Non-treated	Yes	49 (27.2%)	11 (6.1%)	*P* = 0.001 (AP vs. diabetes)
		No	60 (33.3%)	60 (33.3%)	
	Treated	Yes	56 (31.1%)	4 (2.2%)	*P* > 0.05 (AP vs. diabetes)
		No	112 (62.2%)	8 (4.5%)	

Patients were classified as having AP if at least one tooth in the subset under consideration had AP. The P values for various correlations are also indicated.

### Correlation between DM and AP in teeth that were not treated endodontically

The correlation between diabetes and apical periodontitis in non-treated teeth was examined through multiple analyses.

#### Analyzing the distribution of AP by patients

**Figure 2:**
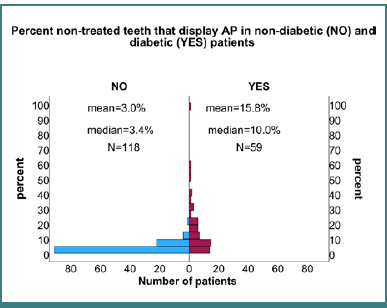
Histogram comparing the percentage of non-treated teeth with apical periodontitis in non-diabetic (left) vs. diabetic groups (right). Means, medians, and patient numbers (n) for both groups demonstrate a higher incidence of AP in patients with diabetes.

A significant correlation was found between diabetes and the prevalence of AP in non-treated teeth. Statistical analysis using the Mann–Whitney test revealed a highly significant difference (*P* < 0.001) in the percentage of teeth with AP per patient between non-diabetic and diabetic groups, as depicted in Figure 2.

#### Analyzing the presence of AP by individual tooth

Further examination using Fisher's exact test indicated a statistically significant correlation between diabetes and the presence of AP in each non-treated tooth (*P* < 0.001), with an odds ratio (OR) of 5.3 (95% CI: 3.9–7.2). This finding suggests that non-treated teeth in patients with diabetes had 5.3 times higher odds of exhibiting AP compared to those in the non-diabetic group.

#### Analyzing the presence of AP by patient

When analyzing the data per-patient basis, a significant correlation between the presence of AP in non-treated teeth and diabetes (*P* = 0.001, Fisher's exact test) was observed, indicating an OR of 4.5 (95% CI: 2.1–9.4). This means that patients with diabetes had 4.5 times higher odds of having at least one non-treated tooth with AP than their non-diabetic counterparts. These findings underscore the pronounced risk of apical periodontitis in patients with diabetes with non-treated teeth, emphasizing the need for diligent dental care and monitoring in the diabetic population.

### Correlation between DM and AP in endodontically treated teeth

The correlation between diabetes and apical periodontitis in treated teeth was analyzed in various contexts, as follows:

#### Analyzing the distribution of AP by patients

When examining the percentage of treated teeth with AP in non-diabetic versus diabetic groups, there was no statistically significant difference (*P* > 0.05, Mann-Whitney test). This indicates that diabetes did not affect the likelihood of AP in treated teeth on a per-patient basis.

#### Analyzing the presence of AP by individual tooth

Similarly, the analysis showed no statistically significant correlation between the presence of AP in each treated tooth and diabetes (*P* > 0.05, Fisher's exact test). This means that, when considering each treated tooth individually, diabetes did not appear to increase the risk of AP.

#### Analyzing the presence of AP by patient

Furthermore, there was no statistically significant correlation between the presence of AP in patients’ treated teeth and diabetes (*P* > 0.05, Fisher's exact test). Thus, at the patient level, diabetes did not significantly impact the occurrence of AP in treated teeth.

### Correlation between DM and AP in treated teeth with adequate RCT

The analysis further explored the correlation between diabetes and apical periodontitis in teeth that received adequate root canal therapy.

#### Analyzing the presence of AP by teeth

When analyzing individual teeth with adequate RCT, there was no statistically significant correlation between AP and diabetes (*P* > 0.05, Fisher's exact test). This indicates that diabetes did not influence the occurrence of AP in adequately treated teeth.

#### Analyzing the distribution of AP by patients

Similarly, there was no statistically significant difference in the percentage of adequately treated RCT teeth with AP between non-diabetic and diabetic groups (*P* > 0.05, Mann-Whitney test). This finding suggests that, on a per-patient basis, diabetes did not affect the likelihood of AP in teeth that received adequate RCT.

### Correlation between AP and adequacy of RCT

Since DM status did not predict the presence of AP in treated teeth, the following investigation delved into the correlation between the adequacy of RCT and the occurrence of AP, both at the individual tooth and patient levels. Through our analysis, significant findings emerged, shedding light on the close relationship between these variables.

#### Analyzing the presence of AP by individual tooth

Firstly, focusing on individual teeth, our study revealed a significant correlation between the inadequacy of RCT and the presence of AP (*P* < 0.001), a connection underscored by the Fisher exact test. The OR was 26.9 (95% CI: 19.0–38.0), indicating that teeth subjected to inadequate RCT had a staggering 26.9-fold increase in the odds of developing AP. Remarkably, this correlation persisted as highly significant (*P* < 0.001) even when controlling for the status of DM.

#### Analyzing the distribution of AP by patient

**Figure 3:**
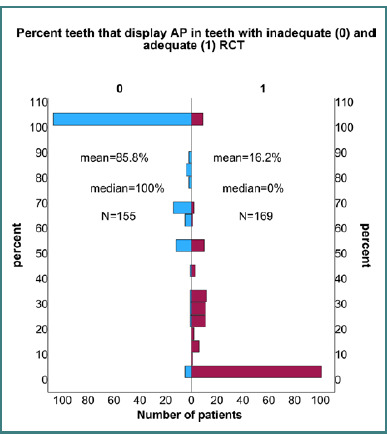
Histogram comparing the percentage of treated teeth that display apical periodontitis for inadequate RCT (left) and adequate RCT (right). Means, medians, and patient numbers (n) for both groups demonstrate a higher incidence of AP in patients with inadequate RCT.

Expanding our investigation to the level of the patient, our analyses continued to confirm these profound insights. A statistically significant contrast emerged in the percentage of teeth displaying AP per patient, depending on whether the RCT of the considered teeth was deemed inadequate or adequate (*P* < 0.001, Mann-Whitney test), as illustrated in Figure 3. The AP percentage means, medians, and the number of patients in each group are also indicated in Figure 3 and further accentuate this disparity, delineating the stark contrast in outcomes based on the adequacy of RCT.

These findings bear profound implications for clinical practice, emphasizing the critical importance of ensuring the adequacy of RCT in mitigating the risk of AP. It underscores the imperative for meticulous attention to detail in endodontic procedures, as suboptimal treatment significantly heightens the likelihood of subsequent AP development, regardless of the patient's diabetic status. Such insights guide clinicians in optimizing treatment protocols, ultimately enhancing patient outcomes and fostering improved oral health.

## DISCUSSION

The results obtained in this study highlight a significant correlation between T2DM and the incidence of apical periodontitis among patients with untreated teeth. In contrast, this correlation was not observed in treated teeth, suggesting that adequate endodontic treatment may reduce the risk of AP in diabetic (as well as non-diabetic) patients.

Data analysis showed that patients with type 2 diabetes were significantly more likely to develop AP in untreated teeth compared to non-diabetic patients. An OR of 5.3 for the presence of AP in untreated teeth and an OR of 4.5 for the presence of AP in patients with untreated teeth suggested a strong association between diabetes and AP. These results are consistent with existing literature emphasizing the increased vulnerability of patients with diabetes to dental infections due to compromised immune responses and impaired microcirculation [8]. Yip *et al*. [18] found that T2DM was independently associated with a significantly higher prevalence of AP (OR = 2.05; 95% confidence interval, 1.73 to 2.43), concluding that T2DM and poorly controlled glycemia are significantly associated with AP.

Although numerous studies have demonstrated a link between AP and diabetes, the precise nature of their relationship remains unclear. Chung *et al*. [19] noted in their study that apical periodontitis tends to be more severe in individuals with poor glycemic control. They found that the prevalence of AP is higher in patients with diabetes and that T2DM and poor glycemic control are significantly associated with AP. Contrary to Chung *et al*. [19], Pérez-Losada *et al*. [5] suggested that no relevant association exists between glycemic control and the prevalence of apical periodontitis or the need for root canal treatment in patients with diabetes.

Regarding endodontically treated teeth, no significant statistical difference was observed between diabetic and non-diabetic patients in our study in terms of AP incidence, both at the level of each treated tooth and at the level of each patient. This suggests that high-quality endodontic treatment can mitigate the negative effects of diabetes on periapical health. Ricucci *et al*. [20] found in their detailed histological study of AP lesions that the healing of periapical tissues is highly dependent on the quality of the root canal treatment, further highlighting that poorly performed treatments lead to persistent AP. Moreover, Ng *et al*. [21] investigated in a systematic review and meta-analysis the factors affecting long-term success rates of root canal treatment, showing that treatment adequacy significantly impacts AP recurrence and healing outcomes. In addition to our findings, Song *et al*. [22] evaluated the factors affecting the success of endodontic treatments and emphasized that procedural quality, including proper cleaning and sealing of canals, is crucial to prevent AP in treated teeth. The findings of our study align with the general principles outlined by Basmadjian-Charles *et al*. [23], who emphasized that long-term success in endodontic treatment is influenced by a combination of factors such as the quality of the root canal therapy, the technique employed, and the patient’s overall health. On the other hand, contrary to our findings, Sisli [9] found a significant association between diabetes mellitus and an increased prevalence of apical periodontitis in root-filled teeth. Moreover, Budreikaitė *et al*. [8] identified a statistically significant relationship between type 2 diabetes mellitus and apical periodontitis in most cases. Additionally, a connection between glycemic control and post-treatment apical periodontitis was observed, which is crucial for managing endodontic patients with T2DM. Preshaw *et al*. [24] offered evidence that maintaining blood glucose within recommended levels can improve the prognosis for AP healing, reinforcing the importance of managing diabetes during endodontic treatment. Therefore, it is recommended to consider that apical periodontitis may occur more frequently in patients with T2DM, and the healing process could be delayed following root canal treatment.

Our results also demonstrated that teeth with inadequate endodontic treatments are much more likely to develop AP (OR = 26.9) than teeth with adequate treatments. This underscores the importance of correctly performed endodontic treatments in preventing AP, regardless of the patient's diabetic status.

According to one study [13], necrotic teeth with periapical periodontitis were generally treated successfully for healing periapical lesions. Endodontic treatment performed in a single visit for necrotic teeth with apical periodontitis could be accomplished in well-controlled diabetic patients. In their study, both groups (control and diabetic) with necrotic teeth and periapical periodontitis were treated successfully regarding the healing of periapical lesions. Endodontic treatments performed in a single visit could be done for diabetic patients if their medical condition is stable and blood glucose levels are controlled. Healing might take up to 1 year to be clearly evident. Moreover, Wang *et al*. [25] found in their study that endodontic therapy led to improvements in apical periodontitis healing, glycemic control, and systemic inflammation in patients with T2DM and/or AP across all groups.

The present study provides important insights into the relationship between diabetes and apical periodontitis, particularly in the context of root canal treatment. One of the most significant findings is the heightened susceptibility of patients with diabetes to AP in untreated teeth, with a 5.3 times greater likelihood compared to non-diabetic patients. This underscores the need for increased vigilance and early intervention in diabetic patients, even in asymptomatic cases. Equally important is the finding that high-quality RCT can significantly reduce the risk of AP in patients with diabetes, aligning their outcomes with those of non-diabetic individuals. This reinforces the critical role that proper cleaning, shaping, and filling of canals and achieving an adequate apical seal play in preventing AP. We also highlighted the detrimental effects of inadequate RCT, which increased the risk of AP by 26.9 times in both diabetic and non-diabetic patients. This underscores the need for practitioners to prioritize RCT adequacy and consider retreatment or specialist referral in complex cases. Moreover, the study emphasizes the importance of frequent follow-ups for patients with diabetes, given their delayed healing potential. Regular radiographic monitoring post-RCT is essential to catch any developing complications early. Finally, the results suggest that comprehensive patient management, including collaboration with medical professionals to control diabetes, is pivotal in ensuring successful endodontic outcomes. Addressing glycemic control before dental procedures and considering prophylactic antibiotics for high-risk patients can improve prognosis.

### Study limitations

Our study has several limitations that need to be acknowledged. Firstly, the cross-sectional design of the study limits the ability to establish causal relationships. Secondly, we did not control for confounding variables such as glycemic control, duration of diabetes, and patient's oral hygiene, which could influence the results. While we collected HbA1c data, the study did not stratify patients based on their level of glycemic control. The cross-sectional study limits our ability to assess the long-term outcomes of root canal treatments in patients with diabetes. A longitudinal design could better evaluate the healing process over time. Additionally, patients were selected from a single private clinic, which may limit the generalizability of the findings. Expanding the study to include larger, more diverse populations to improve generalizability will be our goal for future research.

### Clinical implications and future directions

The results of this study have important clinical implications, emphasizing the importance of high-quality RCT in patients with diabetes, ensuring thorough cleaning, shaping, and sealing of the root canal system, with particular attention to achieving a precise apical seal, ideally within 0-2 mm of the radiographic apex. Additionally, collaboration with medical professionals to ensure proper glycemic control is necessary to schedule more frequent follow-up appointments. Timely intervention can prevent complications and improve long-term results. Future research directions might be oriented toward considering prophylactic antibiotics for high-risk diabetic patients, especially those with poorly controlled glycemia. However, this should be done carefully, balancing the benefits of infection prevention with the risk of contributing to antibiotic resistance. On the other hand, professionals must be involved in educating diabetic patients on the importance of oral hygiene and preventative measures for diabetes management that can empower them to manage their oral health and diabetes better. By focusing on high-quality treatment and proactive management, clinicians can improve the outcomes for this vulnerable patient group.

## CONCLUSION

The study concludes that T2DM significantly increases the risk of apical periodontitis in teeth that were not treated endodontically, with diabetic patients showing a much higher prevalence of AP compared to non-diabetic patients. However, this increased risk does not apply to endodontically treated teeth, even if the root canal treatments (RCT) are performed adequately.

The quality of endodontic treatment is paramount, as inadequate RCT is strongly correlated with a higher incidence of AP, regardless of the patient’s diabetic status. This underscores the critical importance of high-quality endodontic procedures.

In treated teeth, there is no significant difference in the prevalence of AP between diabetic and non-diabetic patients, suggesting that well-executed endodontic treatment can mitigate the adverse effects of diabetes on periapical health. These findings emphasize the necessity for meticulous endodontic treatment in diabetic patients to prevent AP, highlighting that ensuring the adequacy of RCT is vital for successful endodontic outcomes, particularly in individuals with diabetes. Our study underscores the need for an integrated approach to managing diabetes mellitus and oral health to improve clinical outcomes and the patient’s quality of life.

Additionally, further research is needed to understand the underlying mechanisms better and to develop more effective therapeutic approaches for patients with diabetes.

## Data Availability

Further data is available from the corresponding author upon reasonable request.
